# Insufficient comprehensiveness of study collection and leaping to the conclusion

**DOI:** 10.1002/jgf2.437

**Published:** 2021-03-29

**Authors:** Yuki Honda, Toshinori Nishizawa, Keita Morikawa, Tomohisa Oku, Hideki Tsunoda, Yuki Kataoka

**Affiliations:** ^1^ Department of General Internal Medicine Seirei Hamamatsu General Hospital Shizuoka Japan; ^2^ Department of Community Health and Preventive Medicine Hamamatsu University School of Medicine Shizuoka Japan; ^3^ Department of General Internal Medicine St. Luke's International Hospital Tokyo Japan; ^4^ Kameda Medical Center Chiba Japan; ^5^ Oku medical clinic Osaka Japan; ^6^ Department of Family Medicine Shiga University of Medical Science Shiga Japan; ^7^ Hospital Care Research Unit Hyogo Prefectural Amagasaki General Medical Center Hyogo Japan; ^8^ Department of Healthcare Epidemiology Graduate School of Medicine and Public Health Kyoto University Kyoto Japan; ^9^ Systematic Review Workshop Peer Support Group Osaka Japan; ^10^ Department of Respiratory Medicine Hyogo Prefectural Amagasaki General Medical Center Hyogo Japan

To the Editor,

We have read with interest the article by Ota et al.^1^ We applaud the authors’ efforts in conducting a systematic review on the efficacy of shakuyaku‐kanzo‐to (SKT) for the treatment of muscle cramps. However, we have three concerns.

First, the systematic review may have been insufficiently comprehensive.^2^ The authors did not search major literature and clinical trial registration databases, such as Embase, the International Clinical Trials Registry Platform, and ClinicalTrials.gov. Three randomized‐controlled trials were included in their review, but a more extensive search could have yielded a more comprehensive collection of studies. The use of clinical trial registration databases to collect unpublished studies could have reduced publication bias.

Second, the authors should clarify whether they included herbal medicines from countries other than Japan in their review, because herbal medicines such as SKT are frequently used in China and Korea. If herbal medicines from other countries were included, then databases containing Chinese‐ or Korean‐language articles should also have been included. For example, one systematic review on the effect of herbal medicine on post‐stroke spasticity by Cai et al utilized Chinese databases and clinical trial registration websites.^3^ In China, SKT is known as “shao‐yao‐gan‐cao‐tang”; the randomized‐controlled trials reviewed by Cai et al frequently utilized shao‐yao‐gan‐cao‐tang and its two ingredients, “bai shao” and “gan cao,” as interventions. If the authors reviewed studies on Chinese herbal medicines, they should have included “shao‐yao‐gan‐cao‐tang,” “bai shao,” and “gan cao” in their search.

Finally, the authors’ conclusion in the abstract, “SKT might show efficacy in treating muscle cramps in patients with cirrhosis or lumbar spinal stenosis,” was a leap that may lead readers to overestimate the review's results. Of the three trials analyzed, two were at high risk of bias and one was of concern; moreover, a meta‐analysis was not performed because of high heterogeneity. These issues raise doubts about the certainty of the evidence, making it difficult to conclude anything about direction of the effect.

## CONFLICT OF INTEREST

There is no conflict of interest associated with this publication for all authors.
